# Taxonomy of the Genus *Ornativalva* Gozmány, 1955 (Lepidoptera: Gelechiidae) from China †

**DOI:** 10.3390/insects17030293

**Published:** 2026-03-07

**Authors:** Yuqing Zhai, Houhun Li

**Affiliations:** 1 Key Laboratory of Biological Resources and Ecology of Pamirs Plateau in Xinjiang, College of Life and Geographic Sciences, Kashi University, Kashi 844000, China; 13864385798@163.com; 2 College of Life Sciences, Nankai University, Tianjin 300071, China

**Keywords:** Microlepidoptera, Gelechioidea, morphology, taxonomy, review, new species

## Abstract

This paper deals with eleven species of the genus *Ornativalva* Gozmány, 1955 from China based on the specimens collected in the northwest regions of the country. Four species are described as new: *O. artuxiensis* Li, sp. nov., *O. dorsimaculata* Li, sp. nov., *O. longisaccula* Li, sp. nov. and *O. minutispina* Li, sp. nov. Six species are firstly recorded for the Chinese fauna: *O. afghana* Sattler, 1967, *O. levifrons* Sattler, 1976, *O. mongolica* Sattler, 1967, *O. pulchella* Sattler, 1976, *O. sieversi* (Staudinger, 1871) and *O. singula* Sattler, 1967. The female of *O. frontella* Sattler, 1976 is described for the first time. Morphological features of the new species are described and illustrated, and diagnostic characters are given to the species newly recorded for China. A checklist of all the Chinese *Ornativalva* species is included, along with a key to distinguish these species.

## Simple Summary

This study is based on the examination of the specimens collected from nine sampling sites across three provinces (Gansu, Inner Mongolia and Xinjiang) of China during the periods 2006 and 2024 to 2025, with a total of eleven species of the genus *Ornativalva* recognized. These specimens are deposited separately in the Insect Collection of Tianjin Natural History Museum (TJNHM), Tianjin and the Insect Collection of Kashi University (KSU), Xinjiang. The present research supplements the studies on the genus *Ornativalva* conducted from 1991 to 2005, and the relevant investigations in the arid and semi-arid regions. It not only facilitates the comprehensive improvement of the systematic research on *Ornativalva* in China, but also provides fundamental data for the global systematic studies of this genus.

## 1. Introduction

Gozmány established the genus *Ornativalva*, with its type species being *Gelechia plutelliformis* Staudinger, 1859 [1]. Sattler conducted a systematic study on the genus *Ornativalva* and divided 32 species into six species groups: the *cerostomatella* group, the *erubescens* group, the *heluanensis* group, the *ornatella* group, the *plutelliformis* group and the *tamariciella* group [2]. Sattler revised 43 species and one subspecies (*O. mixolitha bipunctella* Sattler, 1967) of *Ornativalva* on a worldwide basis and assigned these species into seven species groups, with the *plicella* group being added on the previous basis [3].

Li et al. carried out a series of taxonomic studies on the genus *Ornativalva* in China and identified a total of 18 species [4,5,6,7,8,9]. Li & Li conducted a cladistic analysis of *Ornativalva* and firstly assigned eight species in China into the system of the seven species groups proposed by Sattler [10]. Bidzilya described two new *Ornativalva* species occurring in the Kyzylkum desert of Uzbekistan [11]. Lee et al. discovered one *Ornativalva* species in the Nearctic region, which had previously only been recorded in the Palearctic region [12]. Nandhini et al. updated the list of Gelechiidae in India, which includes four known *Ornativalva* species [13]. To date, a total of 60 *Ornativalva* species have been recorded in the world [12].

**Figure 1:**
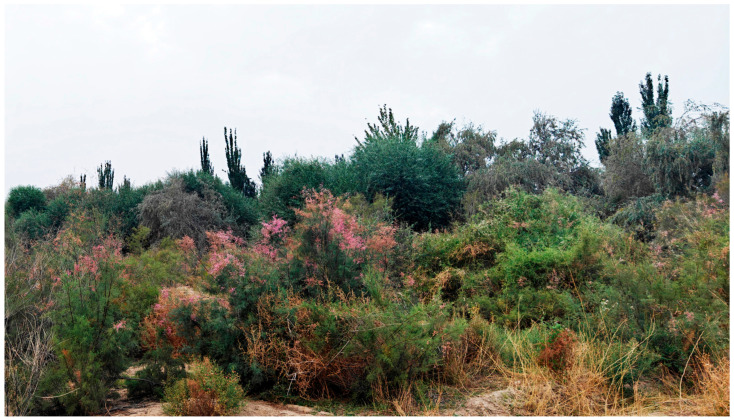
The tamarisk (*Tamarix* sp.) shrub environment of Yilikeqi Township, Yecheng County in Kashi Prefecture, Xinjiang.

Larvae of *Ornativalva* are closely associated with the plant genus *Tamarix* (Tamaricaceae) and one species is additionally recorded to be feeding on *Frankenia* (Frankenaiceae) [3]. Plants of the genus *Tamarix* are an important group of shrubs in sandy wastelands and salinized lands in arid and semi-arid regions (Figure 1). More than 55 species of *Tamarix* are distributed in western Europe, the Mediterranean region, north Africa, India, and northeast and northwest China [14]. *Tamarix* in China includes 18 species and one variety, with 16 species occurring naturally in Xinjiang [15].

Damages to *Tamarix* are mainly caused by some species of Gelechiidae and other families [16]. Domestic research has carried out systematic observations on the annual life history, feeding and reproductive habits of *Ornativalva heluanensis* (Debski, 1913). It has been demonstrated that the larvae of *Ornativalva* are able to develop and grow on *Tamarix* plants, with their populations expanding rapidly and inhibiting the growth of *Tamarix* plants significantly [17]. One species, *O. heluanensis*, feeds on *Frankenia* simultaneously [3]. *Ornativalva* species are widely distributed in the arid and semi-arid regions in northern China, including Gansu, Inner Mongolia, Ningxia, Qinghai, Shaanxi, Tianjin and Xinjiang [9].

The aim of the present paper is to describe four new species, to newly record six species for the Chinese fauna, and to describe the female of *O. frontella* Sattler, 1976 for the first time. A checklist of all the *Ornativalva* species distributed in China is provided, and a key to distinguish these species is included.

## 2. Materials and Methods

Specimens used in this study were collected by using light trapes. Genitalia slides were prepared using the methods of Li [8]. Images of adults and genitalia were taken with Leica M205A stereomicroscope (Leica Instruments (Singapore) Pte Ltd., 12 Teban Gardens Crescent, Singapore), coupled with Leica Application Suite X software (5.1.0.25593).

The type specimens are respectively deposited in the Insect Collection of Tianjin Natural History Museum (TJNHM), Tianjin and Insect Collection of Kashi University (KSU), Xinjiang, China, except stated otherwise.

## 3. Taxonomy

Genus *Ornativalva* Gozmány, 1955

*Ornativalva* Gozmány, 1955: 310. Type species: *Gelechia plutelliformis* Staudinger, 1859.

*Pelostola* Janse, 1960: 188. Type species: *Pelostola kalahariensis* Janse, 1960 [18].

Diagnosis. The genus *Ornativalva* is distinguished by the combinations of the following features: the head of many *Ornativalva* species has a frontal process, the metathorax has paired elongated narrow or short broad scale tufts, and the forewing usually has a W-shaped marking or a longitudinal band; in the male genitalia, the uncus is poorly developed or deeply divided, the gnathos is absent, the valva is divided into two–five branches, and the curved phallus is usually pointed at the apex; in the female genitalia, the papilla anales are rectangular, and the signum consists of a pair of strong spines or teeth, or transverse ridges on an irregularly shaped basal plate.

Notes. Based on Sattler’s system [3], the eleven species treated in this paper can be assigned into the following four species groups: *O. artuxiensis* Li, sp. nov., *O. levifrons* Sattler, 1976, *O. pulchella* Sattler, 1976, and *O. frontella* Sattler, 1976 to the *erubescens* group; *O. minutispina* Li, sp. nov., *O. singula* Sattler, 1967 and *O. sieversi* (Staudinger, 1871) to the *cerostomatella* group; *O. afghana* Sattler, 1967 and *O. mongolica* Sattler, 1967 to the *plutelliformis* group; *O. dorsimaculata* Li, sp. nov., *O. longisaccula* Li, sp. nov. to the *tamariciella* group.

                  **Key to the species of *Ornativalva* in China**

1. Forewing with a W-shaped marking, continuous or discontinuous …………………..2

Forewing without a W-shaped marking ………………………………………………..21

2. Dorsum with a basal streak ………………………………………………………………..3

Dorsum without basal streak ……………………………………………………………..6

3. Head with a short frontal process ([8]: Plate 2, Figure 16) …………………….*O. aspera*

Head without frontal process ……………………………………………………………..4

4. W-shaped marking across fold ……………………………………………………………5

W-shaped marking not across fold (this paper, Figure 3E) …………………*O. singula*

5. Forewing with a white transverse band at 3/4 ([8]: Plate 4, Figure 27) ………*O. sattleri*

Forewing without white transverse band ([8]: Plate 3, Figure 17) ………..*O. basistriga*

6. Harpe present ………………………………………………………………………………7

Harpe absent (this paper, Figure 4A) ………………………………..*O. artuxiensis* sp. nov.

7. Uncus bifid ………………………………………………………………………………….8

Uncus not bifid ………………………………………………………………………………9

8. Uncus with a lateral process, saccus triangular and narrowly pointed at apex (this paper, Figure 4C) …………………………………………………*O. minutispina* sp. nov.

Uncus without lateral process, sacculus semicircular and rounded on anterior margin (this paper, Figure 4E) …………………………………………………………………*O. afghana*

9. Signum with spines ……………………………………………………………………….10

Signum without spine …………………………………………………………………….14

10. Phallus bifurcate apically ([4]: Figure 7) …………………………………………*O. sinica*

Phallus not bifurcate apically ……………………………………………………………..11

11. Valva with a strong and curved apical spine ([8]: Figure 58) ………………….*O. grisea*

Valva without apical spine 12

12. Valva rounded at apex, sacculus subtriangular and narrowed to pointed apex ([8]: Figure 77) ……………………………………………………………………*O. xinjiangensis*

Valva pointed at apex, sacculus subparallel to rounded apex or inflated apically ….13

13. Sacculus subparallel to rounded apex ([8]: Figure 73) …………………*O. plutelliformis*

Sacculus inflated apically ([8]: Figure 79) ……………………………………*O. zepuensis*

14. Signum with ridges/spines ……………………………………………………………….15

Signum without ridge and spine (this paper, Figure 6A) ……*O. dorsimaculata* sp. nov.

15. Head with a truncate frontal process ([8]: Plate 4, Figure 31) …………………*O. zhengi*

Head without frontal process ……………………………………………………………16

16. Sacculus separated from valva …………………………………………………………..17

Sacculus not separated from valva ………………………………………………………19

17. Sacculus almost as long as valva (this paper, Figure 4D) …….*O. longisaccula* sp. nov.

Sacculus distinctly shorter than valva …………………………………………………..18

18. Sacculus more than 3/4 length of valva ([8]: Figure 64) ……………………*O. miniscula*

Sacculus 3/5 length of valva (this paper, Figure 5B) ………………………….*O. pulchella*

19. Valva gradually narrowed distally, with a short apical spine ([8]: Figure 60) …………………………………………………………………………………*O. heluanensis*

Valva sharply narrowed distally, without an apical spine …………………………….20

20. Valva convex ventrobasally, uncus narrowed to acute apex ([9]: Figure 3).…………………………………………………………………………………..*O. acutivalva*

Valva obliquely obtuse ventrobasally, uncus narrowed to obtuse apex ([9]: Figure 7) …………………………………………………………………………………………*O. zonella*

21. Dorsum with a basal streak ………………………………………………………………23

Dorsum without basal streak …………………………………………………………….22

22. Uncus bifid, costal process as long as valva (this paper, Figure 5A) …….*O. mongolica*

Uncus not bifid, costal process longer than valva ([8]: Figure 67) ……*O. novicornifrons*

23. Head without frontal process ([8]: Plate 4, Figure 32) ……………….*O. zhongningensis*

Head with a short frontal process ………………………………………………………..24

24. Sacculus separated from valva …………………………………………………………..26

Sacculus not separated from valva ………………………………………………………25

25. Uncus with apex obtusely rounded, valva gradually narrowed basally (this paper, Figure 4F) …………………………………………………………………………*O. levifrons*

Uncus with apex deeply concave at middle, valva wide and parallel sided basally ([8]: Figure 65) ………………………………………………………………………*O. mixolitha*

26. Sacculus subtriangular ([8]: Figure 57) ……………………………………….*O. frontella*

Sacculus more or less elongate clavate …………………………………………………27

27. Sacculus separated from valva at about basal 1/3 (this paper, Figure 5C) …*O. sieversi*

Sacculus separated from valva at distal 1/3 ([9]: Figures 5 and 6) ………….*O. ornatella*

**Figure 2:**
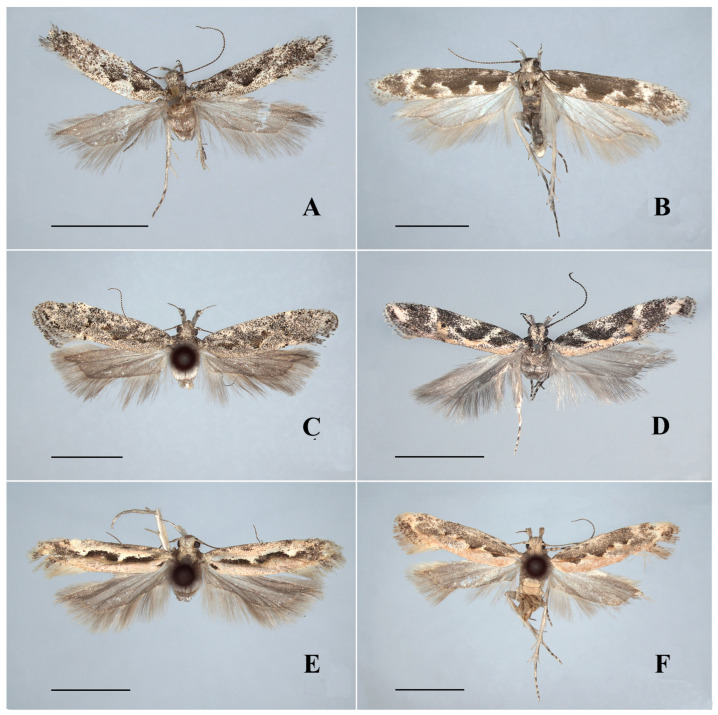
Adults of *Ornativalva* spp. (**A**). *O. artuxiensis* sp. nov., ♂, paratype; (**B**). *O. dorsimaculata* sp. nov., ♂, paratype; (**C**). *O. minutispina* sp. nov., ♂, holotype; (**D**). *O. longisaccula* sp. nov., ♂, holotype; (**E**). *O. frontella* Sattler, 1976, ♂; (**F**). *O. afghana* Sattler, 1967, ♀. Scales = 3.0 mm.

*Ornativalva artuxiensis* Li, sp. nov.
(Figure 2A and Figure 4A)
(LSID urn:lsid:zoobank.org:act:46E5B2FA-2AE3-4F46-8780-A936C4EB76CA)

Type material. Holotype ♂, China, Xinjiang: Kan’are Village, Halajun Township, Artux City, Kizilsu Kirgiz Autonomous Prefecture (40.14° N, 76.78° E), 1584.3 m, 25. V. 2025, leg. XY Zhang et al., slide No. ZYQ25572 (TJNHM).

Paratype. 1 ♂, same data as holotype, slide No. ZYQ25388 (KSU).

Diagnosis. The new species is similar to *O. miniscula*, Li & Zheng, 1995, superficially. It can be distinguished from the latter by the absence of a harpe, and the valva with basal 2/5 three times as wide as distal 3/5 and bent obliquely dorsad distally in the male genitalia. In *O. miniscula*, the harpe is present, and the valva is straight in the basal 3/5 and twice the width of distal 3/5 in the male genitalia [7].

Description. Adult (Figure 2A): Wingspan 10.0–11.0 mm.

Head. Head white, mixed with greyish black, without frontal process. Antenna with scape black on dorsal surface, white mixed with greyish black on ventral surface; flagellum black, interspersed with pale grey rings. Labial palpus with second segment white, mixed with greyish black scales; third segment white, mixed with black.

Thorax. Mesonutom and tegula white, mixed with dark brown. Forewing cream white, with dense dark brown scales; cell with black spot at distal 4/5 and at end respectively; fold with black spot at basal 1/3 and 2/3 respectively; fringe grey, sparsely tipped with brown, paler around tornus. Hindwing and fringe greyish brown.

Abdomen. Male genitalia (Figure 4A): Uncus subrectangular, wider than long. Tegumen trapezoidal posteriorly, with a large, deep anterior emargination extending to posterior 1/3; lateral arm uniform to before slightly narrowed anterior end. Costal process uniformly wide in basal half, narrowed at middle, gradually widened from beyond middle to obtuse apex, setose distally, slightly shorter than valva. Harpe absent. Valva with basal 2/5 inflated, subelliptical, thereafter sharply narrowed and almost uniformly narrow to apex, bent dorsad distally. Sacculus extending to 3/4 length of valva, separated from valva at basal 1/3, uniformly narrow from basal 1/3 to before rounded apex. Saccus broad triangular, as long as uncus. Anellus lobe subovate. Phallus globular in basal 1/3, uniformly narrow in medial 1/3, sharply tapered from distal 1/3 to narrowly rounded apex.

Female unknown.

Distribution. China (Xinjiang).

Etymology. This species is from the type locality ‘Artux’ of Xinjiang.

*Ornativalva dorsimaculata* Li, sp. nov.
(Figure 2B, Figure 4B and Figure 6A)
(LSID urn:lsid:zoobank.org:act:EEF68F09-57CB-4E0E-BF98-5542CCF2EDDE)

Type material. Holotype ♂, China, Xinjiang: Oytag Town, Akto County, Kizilsu Kirgiz Autonomous Prefecture (38.97° N, 75.52° E), 1733 m, 27. VII. 2025, leg. XY Zhang et al., slide No. ZYQ25694 (TJNHM).

Paratypes. 1 ♀, Oytag Town, Akto County, Kizilsu Kirgiz Autonomous Prefecture (38.98° N, 75.51° E), 1801 m, 27. VIII. 2025, leg. A Gulzar et al., slide No. ZYQ25892 (TJNHM); 2 ♂, Xihe Xiu Township, Yecheng County, Kashi Prefecture (36.98° N, 76.71° E), 2881.3 m, 14. VII. 2025, leg. HH Li and SX Wang et al. (KSU).

Diagnosis. The new species is similar to *O. serratisignella* Sattler, 1967 in the male genitalia. It can be distinguished by the fuscous forewing with four white markings; in the male genitalia by the apex-truncate valva without an apical spine and the sacculus separated from the valva at 2/5; in the female genitalia by the signum being a pair of small triangular plates linked by a slender, weakly sclerotized bridge in middle. In *O. serratisignella*, the grey forewing has indistinct brown markings; the valva has an apical spine and the sacculus is separated from the valva at the base; and the signum is a basal plate with a pair of large spines [2].

Description. Adult (Figure 2B): Wingspan 12.5–14.0 mm.

Head. Head white, mixed with brown, without frontal process. Antenna with scape brown, except white at basal 1/4 and distal 1/4; flagellum brown, interspersed with grey rings. Labial palpus with second segment white, mixed with brown; third segment white, covered with brown scales from basal 2/5 to apex.

Thorax. Mesonotum and tegula white, mixed with fuscous. Forewing fuscous, with a white stripe from base obliquely outward to dorsum; costal margin with a white patch extending from distal 1/4 to beyond cell; dorsum with white subtriangular patch at basal 1/3 and 3/5 respectively, both extending crossing fold to cell; fringe grey, mixed with brown. Hindwing light grey, tinged with yellow.

Abdomen. Male genitalia (Figure 4B): Uncus gradually narrowed from base to rounded apex. Tegumen trapezoidal posteriorly, with a large deep anterior emargination; lateral arm uniformly wide to before rounded anterior end. Costal process almost uniformly wide to obtuse apex, setose distally, as long as valva. Harpe slender, digitate, shorter than 1/2 length of valva. Valva triangular, distinctly narrowed from base to truncate apex. Sacculus elongate digitate, separated from valva at 1/2, approx 5/7 length of valva. Vinculum broad triangular, wider than long, with sclerotized lateral edges, 1.4 times length of uncus. Anellus lobe subelliptical. Phallus globular in basal half, uniformly narrow from middle to before pointed apex, curved distally.

Female genitalia (Figure 6A): Papilla anales subquadrate, setose. Apophyses posteriores 2.5 times length of apophyses anteriores. Eighth sternum narrowed toward lateral margin, produced anteromedially. Ductus bursae coiled, longer than corpus bursae. Corpus bursae subelliptical; signum placed posteriorly, composed of two small triangular plates linked by a slender, weakly sclerotized bridge in middle.

Distribution. China (Xinjiang).

Etymology. The specific epithet is derived from the Latin *dorsi-* and *maculatus*, referring to the subtriangular white spots on the dorsum of the forewing.

*Ornativalva minutispina* Li, sp. nov.
(Figure 2C and Figure 4C)
(LSID urn:lsid:zoobank.org:act:07DEF9F0-1842-4454-B06D-ECB6EBB40D8A)

Type material. Holotype ♂, China, Xinjiang: Populus Forest Park, Minfeng County (37.17° N, 82.93° E), 1388 m, 8. VIII. 2024, leg. SX Wang and A Gulzar, slide No. ZYQ24254 (TJNHM).

Paratypes. 2 ♂, same data as holotype, slide Nos. ZYQ24197, ZYQ24246 (KSU).

Diagnosis. The new species is similar to *O. alces* Bidzilya, 2009 and *O. cerva* Bidzilya, 2009 in the forewing pattern and male genitalia. It can be separated from the latter two species by the lateral process of the uncus with two apical spines, the strongly sclerotized valva rounded at the apex, and the costal process exceeding the apex of the valva. In *O. alces* and *O. cerva*, the lateral processes of the uncus lack the apical spines, the weakly sclerotized valvae are pointed at the apices, and the costal processes are as long as the valvae [11].

Description. Adult (Figure 2C): Wingspan 14.0–16.5 mm.

Head. Head white, mixed with greyish brown, with a frontal process. Antenna with scape black except white in basal 1/3 and apex; flagellum black, interspersed with white rings. Labial palpus with second segment white, mixed with greyish black scales; third segment white, with black scales on ventral side.

Thorax. Mesonotum and tegula white, mixed with greyish brown. Forewing cream white, covered with black and fuscous scales; W-shaped marking fuscous, ill-defined, its posterior angles with short black streak at basal 1/3 and 2/3 of fold respectively; cell with black dot of a few black scales at end of W marking and at end of cell; termen with a line of black scales; fringe grey, with brown-tipped scales. Hindwing and fringe brown.

Abodomen. Male genitalia (Figure 4C): Uncus deeply divided into two lobes, each lobe wide at base, uniformly slender from near base to preapex, apex pointed; lateral margin concave inward near base, with a large process arising from middle, which is wide at base, narrowed to apex, with a dorsoapical and a ventroapical spine. Tegumen with a large semicircular anterior emargination, laterally parallel sided to obliquely anterior end. Costal process slender, setose and dilated distally, slightly exceeding apex of valva apically. Harpe triangular. Valva with basal 3/5 widely inflated and parallel sided, abruptly narrowed and uniform from basal 3/5 to apex, forming a slender distal bar rounded at apex. Anellus lobe triangular, setose distally. Saccus triangular, shorter than uncus, narrowly pointed at apex. Phallus globular in basal 1/3, tubular in distal 2/3, with a semicircular notch at apex.

Female unknown.

Distribution. China (Xinjiang).

Etymology. The specific epithet is derived from the Latin *minutus* and *spina*, referring to the two small apical spines of the lateral processes of the uncus in the male genitalia.

*Ornativalva longisaccula* Li, sp. nov.
(Figure 2D, Figure 4D and Figure 6B)
(LSID urn:lsid:zoobank.org:act:BF9BC10C-B615-498E-8A59-C7033BB64879)

Type material. Holotype ♂, China, Xinjiang: Kan’are Village, Halajun Township, Artux City, Kizilsu Kirgiz Autonomous Prefecture (40.14° N, 76.78° E), 1584.3 m, 25. V. 2025, leg. XY Zhang et al., slide No. ZYQ25573 (TJNHM).

Paratypes. 19 ♂ 2 ♀, same data as holotype, slide Nos. ZYQ25391 ♂, ZYQ25399 ♀ (TJNHM) (5 ♂ 1 ♀ in KSU). Inner Mongolia: 1 ♂, Erdaoqiao, Ejin Banner, 927 m, 17. VII. 2006, leg. XP Wang and XF Shi, slide No. ZYQ25674 (TJNHM).

Diagnosis. The new species is similar to *O. zonella* (Chrétien, 1917) in the forewing pattern. It can be separated from the latter in the male genitalia by the elongate trapezoidal uncus, the sacculus slightly shorter than the valva and separated from the valva at basal 1/3, and the phallus is tubular basally. In *O. zonella*, the uncus is broad and short, the sacculus is approx 1/3 the length of the valva and not separated from the valve, and the phallus is dilated spherically [8,9]. The female genitalia are distinguished by having a peanut shell-like signum with a pair of transverse ridges.

Description. Adult (Figure 2D): Wingspan 12.5–14.5 mm.

Head. Head pale yellow, mixed with dark brown, without frontal process. Antenna with scape dark brown on dorsal surface, white on ventral surface; flagellum dark brown, interspersed with white rings. Labial palpus with second segment pale yellow, mixed with black and pale brown scales; third segment black, except white at base and at distal 1/4.

Thorax. Mesonotum dark brown, mixed with cream yellow; tegula dark brown in basal 2/3, cream yellow in distal 1/3. Forewing yellowish white, mixed with yellow scales, with dark brown speckles; costal margin with a dark brown patch from base narrowed obliquely outward to basal 1/3 of fold, second dark brown speckle from basal 1/3 obliquely outward to basal 2/3 of fold, narrowed and curved obliquely up-outward to below anterior margin of cell before middle length of wing, large subquadrate dark brown speckle from distal 2/5 extending crossing anterior angle of cell, wide dark brown band from costal margin near apex straightly downward to above tornus; cell with black spot at distal 1/4 and end, the latter surrounded by yellow scales; irregular earth yellow band extending from base to tornus between below fold and dorsum, with scattered brown scales; fringe grey, some tipped with brown. Hindwing and fringe greyish brown.

Abdomen. Male genitalia (Figure 4D): Uncus elongate trapezoidal, apex concave at middle. Tegumen medially narrow, with a very large anterior emargination; lateral arm narrow, sclerotized along outer margin. Costal process slender basally, inflated distally, setose, shorter than valva, round at apex. Harpe narrow triangular, setose distally, shorter than 1/2 length of costal process. Valva straight, wide at base, narrowed to basal 1/3, then uniformly slender to distal 1/7, distal 1/7 sharply narrowed, spine-like. Sacculus separated from valva at basal 1/3, slightly shorter than valva, wide at base, produced ventrobasally, uniformly slender to before rounded apex, almost as long as valva, not exceeding apex of valva apically. Anellus lobe thumb shaped, setose distally. Vinculum short, triangular, slightly shorter than 1/2 length of uncus. Phallus with basal 1/2 tubular, distal 1/2 tapered to apex.

Female genitalia (Figure 6B): Papilla anales subquadrate, setose. Apophyses anteriores 1/3 length of apophyses posteriores. Eighth sternum widely produced anteriorly. Ductus bursae curly, slightly shorter than corpus bursae. Corpus bursae lageniform, narrowed at middle; signum peanut shell-like, with a pair of transverse ridges, one above middle, the other near posterior end.

Distribution. China (Inner Mongolia, Xinjiang).

Etymology. The specific epithet is derived from the Latin *longus* and the term ‘sacculus’, referring to the long sacculus of the male genitalia.

*Ornativalva frontella* Sattler, 1976
(Figure 2E and Figure 6C)

*Ornativalva frontella* Sattler, 1976: 114. TL: Mongolia. TD: HNHM.

Material examined. China, Xinjiang: 1 ♀, Populus Forest Park, Minfeng County (37.17° N, 82.93° E), 1388 m, 8. VIII. 2024, leg. SX Wang and A Gulzar, slide No. ZYQ24162 (TJNHM).

Diagnosis. Adult (Figure 2E) with wingspan 14.0–16.5 mm. *Ornativalva frontella* is characterized by the forewing with a curved, longitudinal black band interrupted by white patch at above basal 2/3 of fold, and the dorsum with a short black stripe at base. The female genitalia are distinguished by the elongate antrum narrowed anteriorly, and the signum composed of a pair of subrounded basal plates connected by a weakly sclerotized medial bridge.

Female genitalia (Figure 6C): Apophyses anteriores approx 5/8 length of apophyses posteriores. Antrum elongate, slightly narrowed anteriorly. Ductus bursae slender, curly, approx three times as long as corpus bursae. Corpus bursae elliptical; signum located near entrance, consisting of two sclerotized plates connected by a weakly sclerotized medial bridge, each plate with a short spine.

Distribution. China (Xinjiang), Mongolia.

Note. The female of the species is described for the first time to science.

*Ornativalva afghana* Sattler, 1967
(Figure 2F, Figure 4E and Figure 6D)

*Ornativalva afghana* Sattler, 1967: 75. TL: Afghanistan. TD: NHMW.

Material examined. China, Xinjiang: 2 ♂ 4 ♀, National Highway 315, Minfeng County (37.19° N, 82.87° E), 1417.2 m, 3. VIII. 2025, leg. SL Zhang, SY Tang and CJ Zhang, slide Nos. ZYQ25683 ♂, ZYQ25747 ♀, ZYQ25748 ♂ (TJNHM) (1 ♂ 1 ♀ in KSU).

Diagnosis. Adult (Figure 2F) with wingspan 11.5–13.5 mm. *Ornativalva afghana* is characterized by the forewing with posterior half brick red and the cell with two black spots distally. The male genitalia are distinguished by the bifid uncus with each lobe being large subrectangular, the costal process expanded and bent inward distally, the semicircular saccus rounded on anterior margin, and a horn-like apical process of the phallus (Figure 4E). The female genitalia are distinguished by the short and wide apophyses anteriores less than 1/3 the length of the apophyses posteriores, the corpus bursae more than twice the length of the ductus bursae, and the subrhombical signum with a serrate anterior margin and a transverse ridge at the middle (Figure 6D).

Distribution. China (Xinjiang), Afghanistan (Herat), Mongolia.

Note. This species is newly recorded in China.

**Figure 3:**
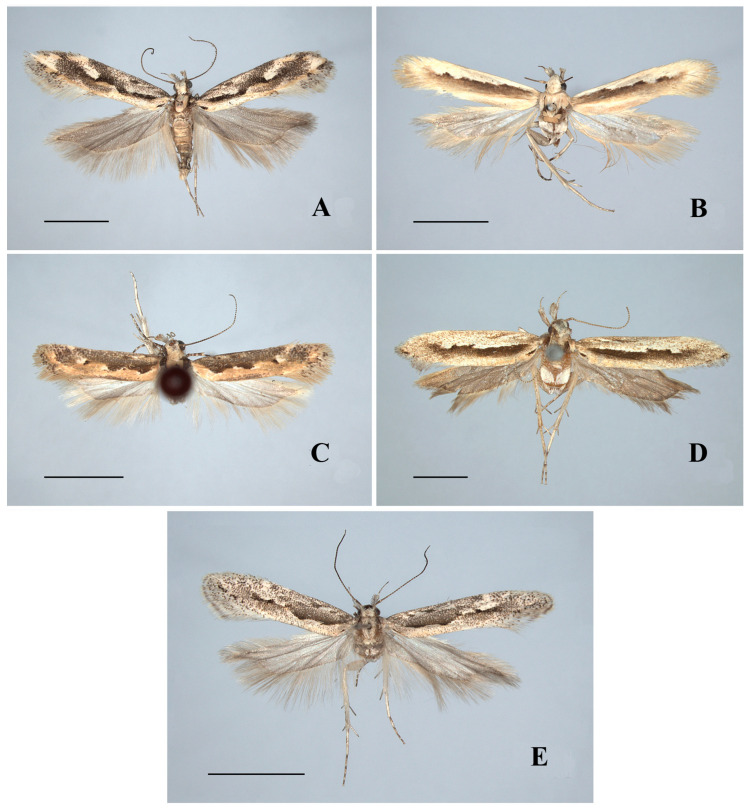
Adults of *Ornativalva*. (**A**). *O. levifrons* Sattler, 1976, ♂; (**B**). *O. mongolica* Sattler, 1967, ♀; (**C**). *O. pulchella* Sattler, 1976, ♂; (**D**). *O. sieversi* (Staudinger, 1871), ♂; (**E**). *O. singula* Sattler, 1967, ♂. Scales = 3.0 mm.

**Figure 4:**
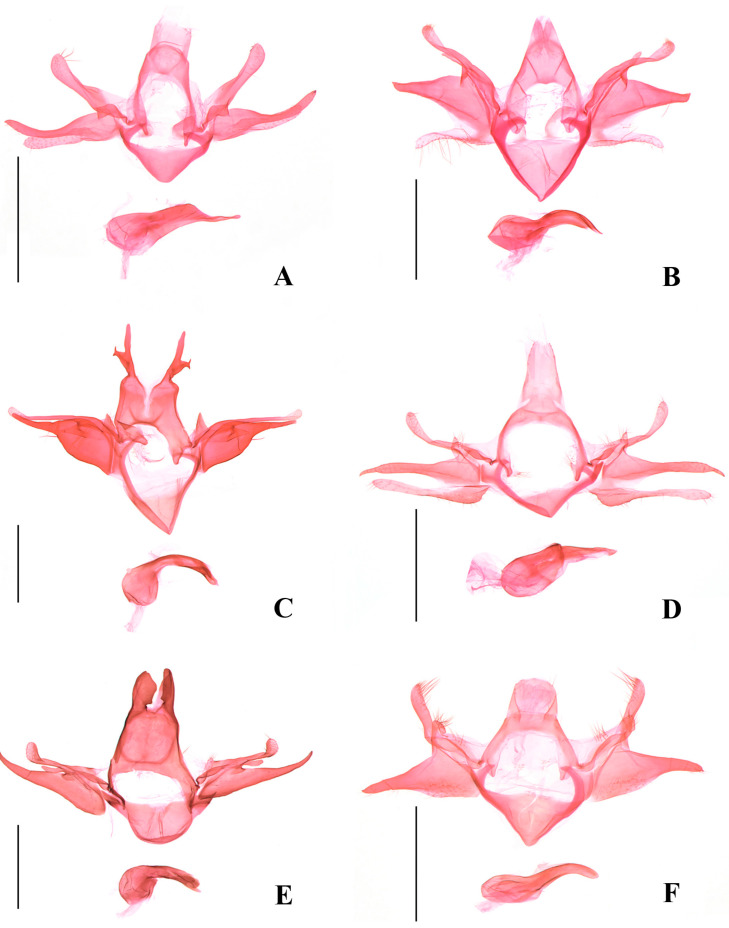
Male genitalia of *Ornativalva* spp. (**A**). *O. artuxiensis* sp. nov., slide No. ZYQ25572, holotype; (**B**). *O. dorsimaculata* sp. nov., slide No. ZYQ25694, holotype; (**C**). *O. minutispina* sp. nov., slide No. ZYQ24254, holotype; (**D**). *O. longisaccula* sp. nov., slide No. ZYQ25573, holotype; (**E**). *O. afghana* Sattler, 1967, slide No. ZYQ25748; (**F**). *O. levifrons* Sattler, 1976, slide No. ZYQ25571. Scales = 0.5 mm.

*Ornativalva levifrons* Sattler, 1976
(Figure 3A, Figure 4F and Figure 6E)

*Ornativalva levifrons* Sattler, 1976: 107. TL: Mongolia. TD: HNHM.

Material examined. China, Inner Mongolia: 1 ♂, Erdaoqiao, Ejin Banner, 927 m, 17. VII. 2006, leg. XP Wang and XF Shi, slide No. OHJ21619 (TJNHM). Xinjiang: 9 ♂, Kan’are Village, Halajun Township, Artux City, Kizilsu Kirgiz Autonomous Prefecture (40.14° N, 76.78° E), 1584.3 m, 25. V. 2025, leg. XY Zhang et al., slide Nos. ZYQ25379, ZYQ25398, ZYQ25571 (TJNHM) (3 ♂ in KSU); 3 ♀, Poplar Forest Park, Minfeng (37.17° N, 82.93° E), 1388 m, 8. VIII. 2024, leg. SX Wang and A Gulzar, slide Nos. ZYQ24163, ZYQ24206, ZYQ24233 (TJNHM) (1 ♀ in KSU).

Diagnosis. Adult (Figure 3A) with wingspan 13.5–21.0 mm. *Ornativalva levifrons* is characterized by the forewing with a curved longitudinal black band running from near base to the end of cell. The male genitalia are distinguished by the broad subquadrate uncus, the slender harpe distally widened to a setose plate, and the setose sacculus not separated from the valva (Figure 4F). The female genitalia are distinguished by the funnel-shaped antrum, and the signum composed of a pair of weakly sclerotized basal plates connected by a narrow bridge (Figure 6E).

Distribution. China (Inner Mongolia, Xinjiang), Mongolia.

Note. This species is newly recorded in China.

**Figure 5:**
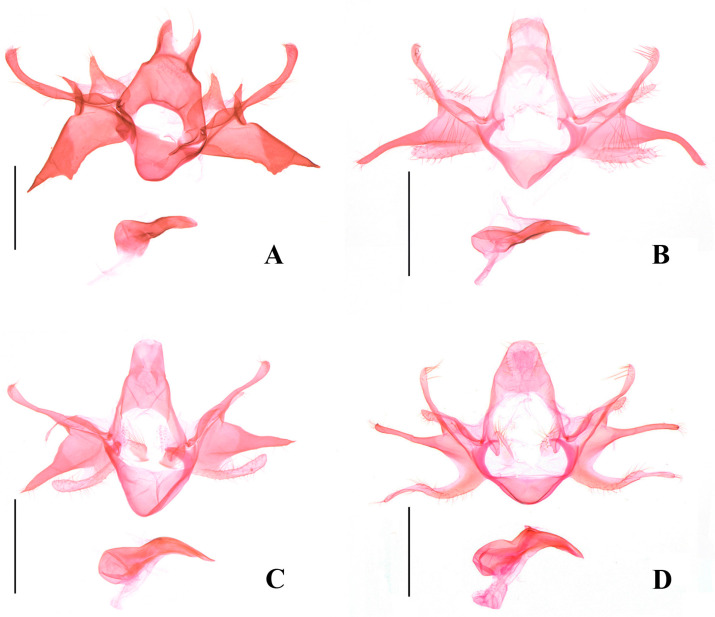
Male genitalia of *Ornativalva* spp. (**A**). *O. mongolica* Sattler, 1967, slide No. ZYQ25684; (**B**). *O. pulchella* Sattler, 1976, slide No. ZYQ24198; (**C**). *O. sieversi* (Staudinger, 1871), slide No. ZYQ25699; (**D**). *O. singula* Sattler, 1967, slide No. ZYQ25393. Scales = 0.5 mm.

*Ornativalva mongolica* Sattler, 1967
(Figure 3B and Figure 5A)

*Ornativalva mongolica* Sattler, 1967: 85. TL: Mongolia. TD: HNHM.

Material examined. China, Gansu: 2 ♂, Poplar Forest, Jinta County (40.01° N, 98.87° E), 1267 m, 6. VIII. 2024, leg. K Lou, Y Liang and SD Zhang, slide No. ZYQ25684 (TJNHM) (1 ♂ in KSU).

Diagnosis. Adult (Figure 3B) with wingspan 11.5–13.5 mm. *Ornativalva mongolica* is characterized by the yellowish white forewing with an irregular longitudinal brown band extending from near base to distal 1/5 along middle of the wing. The male genitalia are distinguished by the bifid uncus with each lobe narrowed to apex, the costal process as long as the valva and bent inward, the valva produced to a narrow triangular dorsodistal process, and the sacculus not separated from the valva (Figure 5A).

Distribution. China (Gansu), Mongolia.

Note. This species is newly recorded in China.

**Figure 6:**
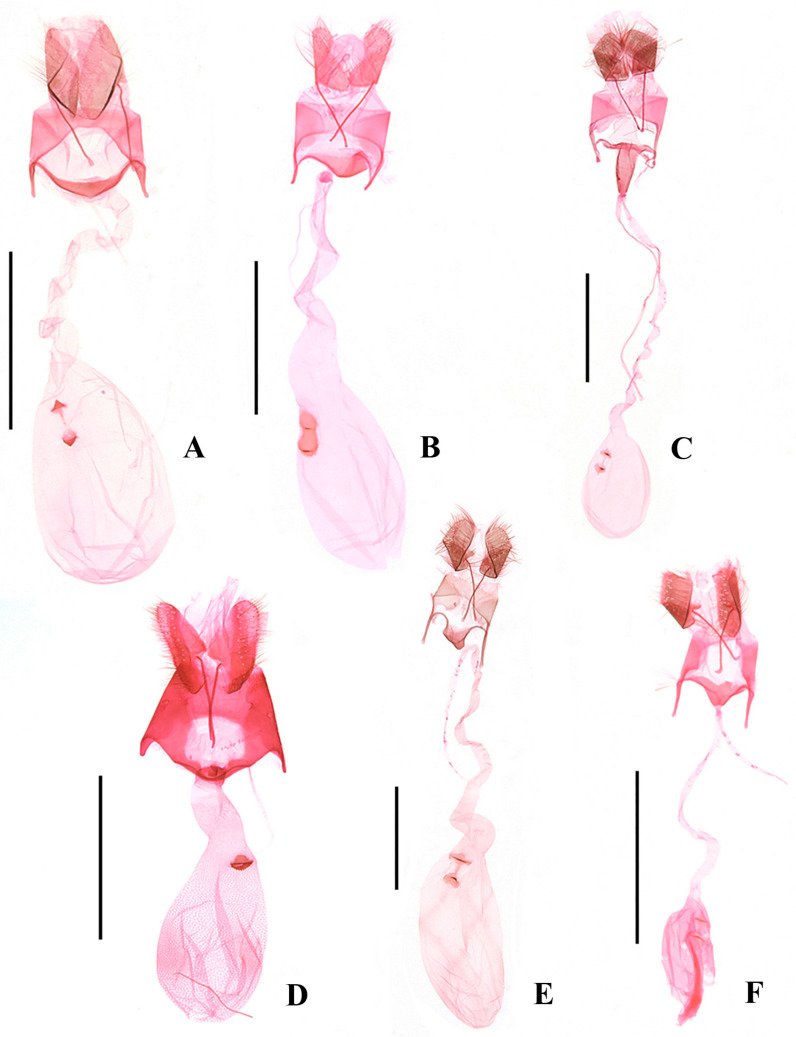
Female genitalia of *Ornativalva* spp. (**A**). *O. dorsimaculata* sp. nov., slide No. ZYQ25892, paratype; (**B**). *O. longisaccula* sp. nov., slide No. ZYQ25399, paratype; (**C**). *O. frontella* Sattler, 1976, slide No. ZYQ24162; (**D**). *O. afghana* Sattler, 1967, slide No. ZYQ25747; (**E**). *O. levifrons* Sattler, 1976, slide No. ZYQ24163; (**F**). *O. pulchella* Sattler, 1976, slide No. ZYQ24199. Scales = 1.0 mm.

*Ornativalva pulchella* Sattler, 1976
(Figure 3C, Figure 5B and Figure 6F)

*Ornativalva pulchella* Sattler, 1976: 110. TL: Mongolia. TD: HNHM.

Material examined. China, Xinjiang: 1 ♂ 1 ♀, East of Jigai Village, Kawake Township, Moyu County (37.62° N, 80.04° E), 1273 m, 1. VIII. 2024, leg. A Gulzar and W Hasan, slide Nos. ZYQ24198 ♂, ZYQ24199 ♀ (TJNHM).

Diagnosis. Adult (Figure 3C) with wingspan 10.5–14.5 mm. *Ornativalva pulchella* is characterized by the forewing with anterior 3/5 dark brown, posterior 2/5 earth yellow with sparse brown scales, and two dark brown spots in basal 2/5 along midline of the wing. The male genitalia are distinguished by the subquadrate uncus parallel sided to obtuse apex, the costal process approx 4/5 the length of the valva, the narrow elongate triangular harpe narrowly rounded at apex, the valva sharply narrowed from middle and thereafter uniformly slender to apex, and the sacculus separated from the valva from basal 2/5 and obtuse at apex (Figure 5B). The female genitalia are distinguished by the strongly sclerotized, funnel-shaped antrum, the signum composed of a pair of weakly sclerotized basal plates and each with a transverse ridge (Figure 6F).

Distribution. China (Xinjiang), Mongolia.

Note. The frontal process of this species exists in some individuals, which is short and truncate; while in some other individuals, it only exhibits as a general outward convexity of the frontal surface.

This species is newly recorded in China.

*Ornativalva sieversi* (Staudinger, 1871)
(Figure 3D and Figure 5C)

*Gelechia sieversi* Staudinger, 1871: 309. TL: S. Russia. TD: MNHU.

*Ornativalva sieversi*: Sattler, 1976: 134.

Material examined. China, Xinjiang: 3 ♂, Riverside, Langru Township, Hetian County (36.90° N, 79.40° E), 1800 m, 3. VIII. 2024, leg. HH Li and W Hasan, slide Nos. ZYQ24167, ZYQ24168; 6 ♂, Homestay near Kizilia Grand Canyon, Kuqa City (42.10° N, 83.04° E), 1487 m, 28. VII. 2025, leg. SX Wang, W Hasan and QY Min, slide No. ZYQ25699 (TJNHM) (1 ♂ in KSU).

Diagnosis. Adult (Figure 3D) with wingspan 17.0–19.5 mm. *Ornativalva sieversi* is characterized by the forewing with a longitudinal dark brown band extending from near base to distal 1/5 and the termen with interrupted black streaks. The male genitalia are distinguished by the distally inflated costal process exceeding the apex of the valva, the subtriangular valva distinctly narrowed from the middle to the spine-shaped apex, and the distally inflated sacculus separated from the valva at about basal 1/3 (Figure 5C).

Distribution. China (Xinjiang), Afghanistan, Iran, S. Russia.

Note. This species is newly recorded in China.

*Ornativalva singula* Sattler, 1967
(Figure 3E and Figure 5D)

*Ornativalva singula* Sattler, 1967: 71. TL: Afghanistan. TD: SMNK.

Material examined. China, Xinjiang: 1 ♂, East Tamarix Forest, Halajun Village, Artux City, Kizilsu Kirgiz Autonomous Prefecture (40.18° N, 76.85° E), 1632.5 m, 25. V. 2025, leg. XY Zhang et al., slide No. ZYQ25393 (TJNHM).

Diagnosis. Adult (Figure 3E) with wingspan 14.5 mm. *Ornativalva singula* is diagnosed by the forewing with two black speckles along the fold, and the cell with two black spots distally. The male genitalia are distinguished by the valva with distal half uniformly slender, the distally sickle-shaped costal process 4/5 the length of the valva, and the sacculus separated from the valva at basal 1/5 and curved in S shape (Figure 5D).

Distribution. China (Xinjiang), Afghanistan, Mongolia.

Note. This species is newly recorded in China.

                  **Checklist of *Ornativalva* species in China**

1. *Ornativalva acutivalva* Sattler, 1976.

*Ornativalva acutivalva* Sattler, 1976: 142. TL: Mongolia (South Gobi Aimak). TD: NHMUK.

Distribution. China (Inner Mongolia), Mongolia.

2. *Ornativalva afghana* Sattler, 1967.

*Ornativalva afghana* Sattler, 1967: 75. TL: Afghanistan (Herat). TD: NHMW.

Distribution. China (Xinjiang), Afghanistan, Mongolia.

3. *Ornativalva artuxiensis* Li, sp. nov.

Distribution. China (Xinjiang).

4. *Ornativalva aspera* Sattler, 1976.

*Ornativalva aspera* Sattler, 1976: 109. TL: Mongolia (South Gobi Aimak). TD: HNHM.

Distribution. China (Ningxia, Xinjiang), Mongolia.

5. *Ornativalva basistriga* Sattler, 1976.

*Ornativalva basistriga* Sattler, 1976: 130. TL: Mongolia (Chovd Aimak). TD: HNHM.

Distribution. China (Inner Mongolia, Ningxia, Xinjiang), Mongolia.

6. *Ornativalva dorsimaculata* Li, sp. nov.

Distribution. China (Xinjiang).

7. *Ornativalva frontella* Sattler, 1976.

*Ornativalva frontella* Sattler, 1976: 114. TL: Mongolia (South Gobi Aimak). TD: HNHM.

Distribution. China (Xinjiang), Mongolia.

8. *Ornativalva grisea* Sattler, 1967.

*Ornativalva grisea* Sattler, 1967: 73. TL: N. Afghanistan (Polichomri). TD: SMNK.

Distribution. China (Inner Mongolia, Xinjiang), Afghanistan.

9. *Ornativalva heluanensis* (Debski, 1913).

*Teleia heluanensis* Debski, 1913: 111. TL: Egypt (Helwan). TD: NHMW [19].

*Teleia frankeniivorella* Chrétien, 1917: 474 [20].

*Teleja oasicolella* Turati, 1924: 161 [21].

*Lita siculella* Mariani, 1937: 9 [22].

*Ornativalva heluanensis*: Sattler, 1967: 38.

Distribution. China (Ningxia, Qinghai, Tianjin, Xinjiang), Algeria, Canary Is., Cape Verde Is., Egypt, Iran, Iraq, Israel, Italy, Libya, Malta, Mongolia, Morocco, Pakistan, Saudi Arabia, S. Russia, Spain, Sudan, Syria, Tunisia, Turkmenistan, Turkey, Yugoslavia.

Biology: Larva on *Frankenia pallida*, *Tamarix nilotica*, *T. tetragyna* [3].

10. *Ornativalva levifrons* Sattler, 1976.

*Ornativalva levifrons* Sattler, 1976: 107. TL: Mongolia (Uburchangaj Aimak). TD: HNHM.

Distribution. China (Inner Mongolia, Xinjiang), Mongolia.

11. *Ornativalva longisaccula* Li, sp. nov.

Distribution. China (Inner Mongolia, Xinjiang).

12. *Ornativalva miniscula* Li & Zheng, 1995.

*Ornativalva miniscula* Li & Zheng, 1995: 333. TL: China (Xinjiang). TD: TJNHM (formerly deposited in NWAFU).

Distribution. China (Xinjiang).

13. *Ornativalva minutispina* Li, sp. nov.

Distribution. China (Xinjiang).

14. *Ornativalva mixolitha* (Meyrick, 1918).

*Phthorimaea mixolitha* Meyrick, 1918: 135. TL: India (Bihar). TD: NHMUK [23].

*Ornativalva mixolitha*: Sattler, 1976: 135.

Distribution. China (Ningxia, Xinjiang), Afghanistan, Algeria, Egypt, India, Iran, Iraq, Mongolia, Morocco, Pakistan, S. Russia, Sudan, Tunisia, Turkey.

15. *Ornativalva mongolica* Sattler, 1967.

*Ornativalva mongolica* Sattler, 1967: 85. TL: Mongolia (East Gobi Aimak). TD: HNHM.

Distribution. China (Gansu), Mongolia.

16. *Ornativalva novicornifrons* Li, 1994.

*Ornativalva novicornifrons* Li, 1994: 78. TL: China (Ningxia). TD: TJNHM (formerly deposited in NWAFU).

Distribution. China (Ningxia).

17. *Ornativalva ornatella* Sattler, 1967.

*Ornativalva ornatella* Sattler, 1967: 78. TL: Afghanistan (Herat). TD: SMNK.

Distribution. China (Inner Mongolia, Xinjiang), Afghanistan, Iran, Mongolia, Romania, S. Russia, Turkey.

18. *Ornativalva plutelliformis* (Staudinger, 1859).

*Gelechia plutelliformis* Staudinger, 1859: 239. TL: Spain (Cadiz). TD: MfN [24].

*Alucita olbiaella* Millière, [1861]: 193 [25].

*Hypsolophus siewersiellus* Christoph, 1867: 239 [26].

*Gelechia sinuatella* Walsingham, 1904: 223 [27].

*Ornativalva plutelliformis*: Sattler, 1967: 72.

Distribution. China (Inner Mongolia, Xinjiang), Afghanistan, Algeria, Arabia, Canary Is., Cyprus, Egypt, Hungary, Iran, Iraq, Israel, Italy, Jordan, Lebanon, Libya, Madeira, Morocco, Pakistan, Romania, S. France, S. Russia, Spain, Sudan, Syria, Tunisia, Turkey, Yugoslavia.

Biology: Larva on *Tamarix africana*, *T. canariensis*, *T. gallica*, *T. laxa*, *T. pallasii*, *T. parviflora* [3].

19. *Ornativalva pulchella* Sattler, 1976.

*Ornativalva pulchella* Sattler, 1976: 110. TL: Mongolia (South Gobi Aimak). TD: HNHM.

Distribution. China (Xinjiang), Mongolia.

20. *Ornativalva sattleri* Li & Zheng, 1995

*Ornativalva* sp. 4 Sattler, 1976: 119.

*Ornativalva sattleri* Li & Zheng, 1995: 332. TL: China (Xinjiang). TD: TJNHM (formerly deposited in NWAFU).

Distribution. China (Xinjiang), Mongolia.

21. *Ornativalva sieversi* (Staudinger, 1871).

*Gelechia sieversi* Staudinger, 1871: 309. TL: S. Russia (Krasnoarmeysk). TD: MNHU [28].

*Ornativalva sieversi*: Sattler, 1976: 134.

Distribution. China (Xinjiang), Afghanistan, Iran, Russia.

22. *Ornativalva singula* Sattler, 1967.

*Ornativalva singula* Sattler, 1967: 71. TL: Afghanistan (Herat). TD: SMNK.

Distribution. China (Xinjiang), Afghanistan, Mongolia.

23. *Ornativalva sinica* Li, 1991.

*Ornativalva sinica* Li, 1991: 88. TL: China (Shaanxi). TD: TJNHM (formerly deposited in NWAFU).

Distribution. China (Inner Mongolia, Ningxia, Shaanxi, Tianjin).

24. *Ornativalva xinjiangensis* Li, 1991.

*Ornativalva xinjiangensis* Li, 1991: 89. TL: China (Xinjiang). TD: TJNHM (formerly deposited in NWAFU).

Distribution. China (Xinjiang).

25. *Ornativalva zepuensis* Li & Zheng, 1995.

*Ornativalva zepuensis* Li & Zheng, 1995: 332. TL: China (Xinjiang). TD: TJNHM (formerly deposited in NWAFU).

Distribution. China (Inner Mongolia, Ningxia, Qinghai, Xinjiang).

26. *Ornativalva zhengi* Li, 1994.

*Ornativalva zhengi* Li, 1994: 77. TL: China (Ningxia). TD: TJNHM (formerly deposited in NWAFU).

Distribution. China (Ningxia).

27. *Ornativalva zhongningensis* Li, 1994.

*Ornativalva zhongningensis* Li, 1994: 80. TL: China (Ningxia). TD: TJNHM (formerly deposited in NWAFU).

Distribution. China (Ningxia).

28. *Ornativalva zonella* (Chrétien, 1917).

*Teleia zonella* Chrétien, 1917: 474. TL: Tunisia (Gafsa). TD: MNHN.

*Teleia cimelion* Amsel, 1935: 210 [29].

*Ornativalva iranella* Sattler, 1967: 44.

*Ornativalva zonella*: Sattler, 1976: 105.

Distribution. China (Inner Mongolia, Xinjiang), Algeria, Iran, Israel, Saudi Arabia, Tunisia.

## 4. Discussion

This study focuses on the taxonomy of the genus *Ornativalva* in China, with four species described as new and six species newly recorded for the Chinese fauna. Counting the previously recorded eighteen species, a total of twenty-eight species have been recorded in China. These findings have highlighted the species diversity of the genus in the northwestern region of China [8]. India, the neighbouring country of the southwestern region of China, has four described species [13]. It can thus be inferred that this genus should also be distributed in southwestern China, especially in Yunnan and southern Xizang. The results of this study also indicate that there may be some unknown *Ornativalva* species in arid, semi-arid, and remote areas.

Among the new species recognized in this study, *O. minutispina* Li, sp. nov. possesses a highly developed uncus and is similar to *O. alces* Bidzilya, 2009 and *O. cerva* Bidzilya, 2009 described by Bidzilya from Uzbekistan [11]. The most significant difference is that the uncus of the new species bears a ventral and a dorsal spine in its lateral process. Accordingly, this species is assigned to the *cerostomatella* group.

*Ornativalva pulchella* Sattler, 1976 is reported herein as a newly recorded species of China, which was collected from Moyu County, southern Xinjiang. Observations revealed that the head of the individuals collected in Xinjiang lacks a distinct frontal process, while individuals recorded from Mongolia possess a short, truncate frontal process [3]. Whether this variation is caused by the microenvironmental change requires further biological investigation of the species in the two regions.

## Data Availability

All data are contained within the article.

## Abbreviations

HNHM	Hungarian Natural History Museum, Budapest, Hungary.
KSU	Insect Collection of Kashi University, Xinjiang, China.
MNM	Magyar Nemzeti Museum, Budapest, Hungary.
MNHN	Muséum National d’Histoire Naturelle, Paris, France.
MfN	Museum für Naturkunde der Humboldt-Universität, Berlin, Germany.
NHMUK	Natural History Museum, Department of Zoology, London, UK.
NHMW	Naturhistorisches Museum, Wien, Austria.
NWAFU	Northwest Agriculture and Forestry University, Shaanxi, China.
TD	Type depository.
TL	Type locality.
TJNHM	Insect Collection of Tianjin Natural History Museum, Tianjin, China.

## References

[1] Gozmány L. (1955). Notes on some Hungarian Gelechioidea and Coleophoridae. Ann. Hist.-Nat. Musei Natl. Hung..

[2] Sattler K. (1967). Die Gattungen *Ornativalva* Gozmány und *Horridovalva* gen. n. (Lepidoptera, Gelechiidae). Beiträge Zur Naturkundlichen Forsch. Südwestdeutschland.

[3] Sattler K. (1976). A taxonomic revision of the genus *Ornativalva* Gozmány, 1955 (Lepidoptera: Gelechiidae). Bull. Br. Mus. (Nat. Hist.) Entomol..

[4] Li H.H. (1991). A study on the Chinese species of *Ornativalva* Gozmány (Lepidoptera, Gelechiidae). Entomotaxonomia.

[5] Li H.H., Li Y.Y. (1992). Two new species *Ornativalva* Gozmány newly recorded from China (Lepidoptera: Gelechiidae). J. Northwest For. Coll..

[6] Li H.H. (1994). Three new species and one new record of the genus *Ornativalva* Gozmány (Lepidoptera: Gelechiidae) from China. Entomol. Res..

[7] Li H.H., Zheng Z.M. (1995). Three new species and three new records of the genus *Ornativalva* from Xinjiang, China (Lepidoptera: Gelechiidae). Zool. Res..

[8] Li H.H. (2002). The Gelechiidae of China (I) (Lepidoptera: Gelechiidae).

[9] Li H.H., Li Z.Q., You P. (2003). The genus *Ornativalva* from Inner Mongolia and Tianjin of China (Lepidoptera: Gelechiidae). Acta Zootaxonomica Sin..

[10] Li Z.Q., Li H.H. (2005). Cladistic biogeography of the genus *Ornativalva* Gozmány (Lepidoptera: Gelechiidae). Acta Entomol. Sin..

[11] Bidzilya O.V. (2009). Two new remarkable species of the genus *Ornativalva* Gozmány, 1955 (Lepidoptera, Gelechiidae) from Uzbekistan. Entomofauna.

[12] Lee S., Watkinson I., Vargo J. (2017). *Ornativalva erubescens* (Walsingham) (Lepidoptera: Gelechiidae) Introduced in North America. J. Lepid. Soc..

[13] Nandhini D., Shashank P.R., Joshi R., Reddy K.M. (2024). A catalogue of Indian Gelechiidae Stainton, 1854 (Lepidoptera: Gelechioidea). Zootaxa.

[14] Heywood V.H., Brummit R.K., Culham A., Seberg O. (2007). Flowering Plant Families of the World.

[15] Yang W.K., Zhang D.Y., Zhang L.Y., Yin L.K. (2004). Study on ecological types and Habitat similarity of *Tamarix* L. in Xinjiang. Arid. Land Geogr..

[16] Pang X.A., Jiang X., Wang J.X., Yang M.L. (2008). Recent progresses in the research of *Tamarix* L. in China. J. Tarim Univ..

[17] Li B.P., Ma X.X., DeLoach J. (2004). *Ornativalva heluanensis* Debski (Lepidoptera: Gelechiidae), a potential biocontrol agent of Saltcedar. Chin. J. Biol. Control..

[18] Janse A.J.T. (1960). Moths of South Africa 6, Gelechiadae. Moths S. Afr..

[19] Debski B., Andres A. (1913). Verzeichnis der bis jetzt in Aegypten beobachteten Schmetterlinge. Bull. Société Entomol..

[20] Chrétien P. (1917). Contribution a la connaissance de lépidoptères du nord de l’Afrique. Notes biologiques et critiques. Ann. Société Entomol. Fr..

[21] Turati E. (1924). Spedizione Lepidotterologica in Cirenaica 1921–1922. Atti Della Soc. Ital. Delle Sci. Nat..

[22] Mariani M. (1937). Nuove specie e forme di Lepidopteri di Sicilia ed un nuovo parassita degli agrumi. Soc. Di Sci. Nat. Econ.-Iche Di Palermo.

[23] Meyrick E. (1918). Exotic Microlepidoptera. Vol. II. Exot. Microlepidoptera.

[24] Staudinger O. (1859). Diagnosen nebst kurzen beschreibungen neuer Andalusischer Lepidopteren. Stettin. Entomol. Ztg..

[25] Millière P. (1861). Iconographie et Description de Chenilles et Lépidoptéres. Icon. Descr. Chenilles Lépidoptères..

[26] Christoph H. (1867). Beschreibung einiger neuer Schmetterlinge aus der Umgegend von Sarepta. Stettin. Entomol. Ztg..

[27] Walsingham L. (1904). Algerian Microlepidoptera. Entomol. Mon. Mag..

[28] Staudinger O. (1871). Beschreibung neuer Lepidopteren des europäischen faunegebiets. Berl. Entomol. Z..

[29] Amsel H.G. (1935). Zur Kenntnis der Microplepidopterenfauna des südlichen Toten-Meer-Gebietes, nebst Beschreibung neuer palästine- nsischer Macro-und Microlepidoptera. Veröffentlichungen Dtsch. Kolonialen Übersee-Mus..

